# Designing Innovative Assistive Technology Devices for Tourism

**DOI:** 10.3390/ijerph192114186

**Published:** 2022-10-30

**Authors:** Marek Zabłocki, Bogdan Branowski, Przemysław Kurczewski, Jarosław Gabryelski, Maciej Sydor

**Affiliations:** 1 Institute of Transport, Faculty of Civil and Transport Engineering, Poznan University of Technology, 60-965 Poznan, Poland; 2 Digitouch sp. z o.o., ul. Różana 6, 62-002 Suchy Las, Poland; 3 Department of Woodworking and Fundamentals of Machine Design, Faculty of Forestry and Wood Technology, Poznan University of Life Sciences, 60-637 Poznan, Poland

**Keywords:** people with disabilities, a person with a disability, technological innovation, design, physical activity, rehabilitation, active tourism

## Abstract

Active tourism improves human health and well-being regardless of age or disabilities. The paper analyses and describes current issues concerning the tourism of people with disabilities. The starting point is the currently insufficient availability of tourist offers for individuals with considerable motor dysfunctions. One of the causes for these limitations stems from deficiencies in transport means for people with disabilities. It was found that for a disabled passenger using public transport, it is crucial to consider its accessibility in the context of the entire transport system. Another cause is the limited popularity of innovative, atypical assistive equipment for people with disabilities. Those insights point out that novel assistive technologies need to be developed, as it is necessary to more effectively support the activity of people with disabilities in all areas of life, including tourism, as this enhances their social rehabilitation. This paper indicates the needs and describes and analyses examples of own original, innovative devices supporting the areas mentioned above of activity for people with disabilities. These analyses resulted in developing an algorithm to design innovative equipment, considerably expanding the tourism potential of people with motor disabilities. This design process focuses on the needs of people with disabilities and facilitates the development of novel classes of assistive technologies, thus promoting new areas of activity for all.

## 1. Introduction

According to the World Health Organization (WHO), there are 1 billion people with disabilities worldwide, representing 15% of the global population. Among them, 2 to 4% experience considerable problems with daily functioning. Additionally, it must be remembered that the number of people with disabilities is growing continuously, and as indicated by the WHO, this trend is associated, among other things, with population aging and the rapid increase in the prevalence of chronic diseases [1]. Compared to previous periods, people with disabilities also suffered much more from limitations in their functioning during the COVID-19 pandemic [2]. As has also been stressed by the WHO, in many regions around the world, health care for disabled patients is substandard and underfinanced, so there is a pressing need to expand the range of rehabilitation services [3] to ensure continuous rehabilitation and maintain positive effects of medical rehabilitation initiated at the beginning of treatment while patients are still in hospital [4].

According to the model proposed by professor Wiktor Dega, medical rehabilitation needs to be common, early, comprehensive, and continuous. In 1970, the WHO accepted and recommended the model developed by W. Dega [5]. The rehabilitation process meeting present-day requirements should comprise comprehensive activities, including medical and social rehabilitation maintained over an extended period [6,7]. Medical rehabilitation is provided inpatient or outpatient, whereas social rehabilitation includes environmental therapy (milieu therapy or community therapy) and occupational therapy. These two primary forms of rehabilitation include physical and psychological rehabilitation [8]. Both types of rehabilitation require further efforts to search for new procedures and techniques to enhance their effectiveness.

Numerous studies have shown that physical impairments are the most common cause of disabilities. For example, investigations made by Okoro et al. [9] show that in the U.S. population over age of 18, physical impairments were the most widespread type of disability (13.7%), followed by cognitive dysfunctions (10.8%), hearing (5.9%), and visual impairment (4.6%). The prevalence of hearing disabilities and mobility impairments was greater among the elderly, whereas the frequency of cognitive impairment was highest among the middle-aged (11.9%) and young adults (10.6%), while it was lowest among older people (9.5%). It also results from a study by Courtney-Long [10] in which the most commonly reported types of limitations were mobility and cognitive disabilities. Further studies on the subject showed that among people who report severe impairments, 46% indicate motor disability, 39% impaired problem-solving or concentration capacity, 26% report hearing and 21% visual impairment, while 43% report more than one type of impairment [11]. The sources of the global prevalence of diseases and injuries worldwide point main causes of disability among people aged 10–49 years. These are injuries related to traffic accidents, communicable diseases (infectious diseases), and non-communicable diseases such as diabetes, arthritis, and depression. Cardiovascular disease was the top-ranked cause of disability in the 50–74-year and 75-year and older age groups [12,13]. Lifestyle choices and personal predispositions such as obesity, physical inactivity, and the use of stimulants such as tobacco, alcohol, and illicit drugs are also significant factors causing disability.

The group of people with motor disabilities is not homogeneous, and its diversity is derived from the character and underlying causes of these impairments. Generally, mobility impairments are caused by innate developmental defects, disease, unhealthy lifestyle (at present, it is frequently addiction to stimulants, obesity, and uncontrolled and extreme physical activity), and biomechanical overload in the case of manual workers. A disability may also be caused by various accidents, the risk of which increases with population aging [14], or it may be a consequence of military conflicts. It was also observed that starting from the age of 50+ or even 40+, procedures different from those typically applied in the case of young people are needed, aiming at rehabilitation after injury or disease and maintenance of psycho-physical health over a more extended period. Social disability (non-participation in various forms of life activity) is closely related to a lack of adaptation of the living environment to a given person’s needs in terms of infrastructure and assistive technology (AT). Unfortunately, frequent causes of mobility problems are still related to inappropriately designed or used individual technical equipment [15,16]. Ignorance and disregard for the unique needs of persons with disabilities when formulating requirements imposed on equipment designed specifically for them, combined with the inadequate knowledge of specialists in this field, generally limit the potential for normal functioning of individuals with disabilities following the concept of independent living [17]. According to the UN Convention on the rights of people with disabilities, people are disabled due to external barriers rather than individual limitations [18]. Thus, eliminating these external barriers is an effective method of reducing disabilities.

Tourism is a form of physical and social activity performed outdoors. Tourism requires mobility and therefore supports physical activity. Movement improves human health regardless of the age or potential disabilities of individuals. Exercise associated with tourism, particularly when it is moderate, in combination with other factors that affect the human organism, has a beneficial influence on human physiology [19,20]. Physical activity serves as an impulse that stimulates the central nervous system, which also improves cognitive functions [21].

In addition, it increases the aerobic fitness of the organism and the efficiency of the circulatory system, while it also improves smooth muscle fitness, the function of abdominal organs, and the pelvis, which in turn prevents gait and balance disorders. Physical activity improves blood flow efficiency, reduces the risk of blood density and clotting in blood vessels, normalizes blood glucose levels, reduces the concentration of low-density lipoproteins (LDL), and increases the concentration of high-density lipoproteins (HDL), which improves the immune system function, accelerates metabolism, promotes mental well-being, as well as enhances physical fitness [22]. These positive changes in the function of the human organism associated with physical activity may also be recorded by governmental bodies as a reduction in demand for rehabilitation among individuals with low physical activity.

Physical activity, in combination with rehabilitation training, promotes greater daily life fitness, i.e., capacity to perform various activities of daily living (ADLs) (np. functional mobility such as the ability to walk or use a wheelchair, get in and out of bed, and a chair; dressing, grooming, self-feeding) even at such extensive disabilities such as paraplegia or tetraplegia. Systematic rehabilitation training as part of rehabilitation was implemented in various countries many years ago when promoting the concept of the so-called active rehabilitation (in Sweden in 1976 and in Poland in 1988 [23]). The active rehabilitation movement has contributed to searching for new activity areas for people with disabilities.

**Figure 1:**
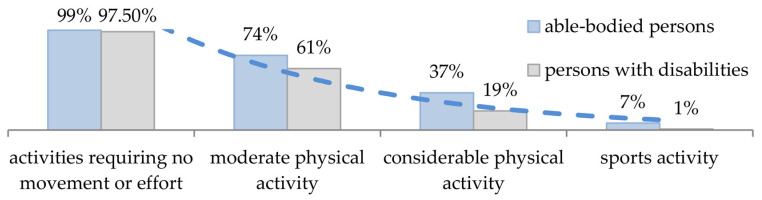
The percentage shares of various leisure activities (based on GUS data [24]).

Unfortunately, physical activity is not very common among people with motor dysfunctions. Analyses by the Central Statistical Office (Statistics Poland) [24] provide information on the physical activity of people with disabilities in terms of the various forms of leisure activity in Poland (Figure 1). These include:

- Activities requiring practically no movement or exercise: reading, watching TV, listening to the radio, and other similar activities;
- Moderate physical activity: walking, gymnastics, or other forms of relatively moderate exercise;
- Considerable physical activity: swimming, other recreational sports, seasonal gardening, and other outdoor leisure activity;
- Sports activity: intensive training (e.g., at a gym) or competitive sports.

It results from the data given in Figure 1 that the greater the effort during physical activity, the lesser the share of participating people with disabilities compared to non-disabled individuals. In the case of very limited or limited activity, the differences in both groups are slight; for considerable physical activity and sports activity, the difference is more significant. However, this conclusion seems relatively ambiguous. It may be asked whether such a situation may result from a lack of willingness to actively participate in specific activities, organizational and financial limitations, or a lack of available technical equipment adapted to variable fields of use and weather conditions. It is also worth pointing out that some people with disabilities have contraindications to strenuous physical exertion, so they require specific assistive devices. All of these causes may be involved simultaneously; however, experience shows that the primary and, at the same time, most challenging limitation is related to the inaccessibility of appropriate equipment for individual users, which would promote active tourism for people with disabilities.

This paper aims to propose an algorithm that supports the design process of innovative assistive technology devices, expanding the areas of participation of individuals with motor disabilities in active tourism.

## 2. Diagnosis of Needs: Challenges in the Field of Active Tourism for People with Disabilities

### 2.1. Tourism of People with Disabilities

According to the Polish Tourist and Sightseeing Society PTTK, the concept of tourism for people with disabilities is defined as: “intentional, purposeful physical activity adapted to individual needs, performed in various forms, closely related to sightseeing activity. It is a form of social rehabilitation for people with disabilities. This activity aims to maximize physical, mental, social, and occupational fitness as well as adaptation to normal life. Tourism for people with various disabilities is a recreation and a means of therapy and education” [25].

The presentation of specific information on travel for people with disabilities is impeded by a lack of available statistics [25], while studies on tourist preferences are performed rarely and to a limited extent, preventing reliable generalizations on the subject. According to the scarce data in the literature, it may be stated that, e.g., in Poland, people with disabilities around 15 years ago traveled more than three times less often compared to non-disabled individuals, while their participation in foreign travel was 14 times lower [26]. For example, the first specialist travel agency for people with disabilities was founded in Poland in 2009, and even today, this industry section is seriously underdeveloped.

The PTTK report shows that the share of people with disabilities in tourism is 1.8%. In comparison to able-bodied tourists, this number is extremely low. Among the various forms of active tourism of people with disabilities, mountain and lowland hiking predominates (64% share in the entire activity of people with disabilities), followed by cycling and kayaking (approx. 14%). Horse riding (4%), sailing (1.7%), motorsports (1.6%), skiing (0.3%), and diving (0.02%) are rarely practiced [25,26].

Bauer indicated four factors that hinder the development of tourism for people with disabilities: intrapersonal, interpersonal, economic, physical, and attitudinal barriers [27]. Intrapersonal factors include physical, sensory, psychological, and cognitive limitations to contemplating travel. Interpersonal barriers manifest in disturbed interactions with travel companions as well as strange transportation, accommodation, and other service providers. Problems related to the execution of the need for leisure time activities by people with motor disabilities also result from physical limitations, observed first of all in non-urbanized areas when these individuals participate in tourist activities (e.g., moving on trails, in open areas, in sandy terrain [28]) or, for example, in amateur practice of various sports disciplines. For many years, such forms of activity were very poorly developed [29] due to a lack of available technical means of transport in difficult terrain or no technical equipment dedicated to different sports disciplines being produced. Execution of amateur sports or tourism activities is directly associated with the gradual rehabilitation process for people with disabilities, constituting a framework for social rehabilitation. Additionally, adapting such equipment is even more complicated because, compared to the past, tourists’ present-day behavior and needs have significantly changed [30]. Economic factors are significant. Travel and accommodation costs are usually higher for people with a disability. Not all transport methods are available, and suitable accommodation is usually only available in more expensive locations. Social attitudes produce attitudinal barriers for disabled tourists. Negative attitudes of other tourists or passengers are associated with the stereotype that the presence of disabled counterparts may disturb their otherwise carefree and ideal holiday environment.

### 2.2. Satisfying the Transport Needs of People with Disabilities

Transport is an indispensable element of every tourist enterprise. Individuals with mobility impairment are at risk of being discriminated against in terms of public transport accessibility, especially when the journey takes place in a new, unfamiliar area, and it is logistically complicated when, for example, it requires a transfer to another means of transport, e.g., from a railway to a road or an air transport. Discrimination refers not only to the lack of adaptation of specific means of transport to special needs but also to the complexity of regulations and detailed rules of transport use. The movement of disabled people within the entire public transport system is considerably hindered by the continuing need to search for information on the availability of adapted transport means and regulations concerning the transport of individuals with significant disabilities, moving in wheelchairs. Frequently the intention to travel on a train or airplane needs to be reported much earlier, as a result, it is difficult to make flexible travel plans or introduce impromptu changes to the itinerary. This hinders the accessibility of the public transport system as a whole. Passengers typically use the entire system rather than only one means of transport and when a section of the itinerary may not be covered or when finding information required when traveling is too time-consuming, they simply either decide not to travel or choose an individual means of transport (a car) [31].

For most people with disabilities, inaccessible transport harms their everyday life. Problems with accessibility, particularly in the case of public transport systems, may considerably reduce their employability and limit the potential for social inclusion of disabled people [31], while particularly hindering tourism of individuals with disabilities.

The needs related to mobility and transport for people with disabilities change with age [32]. Many social factors also affect the need for mobility [33]. The inclusive tourism sector appears to be one of the primary beneficiaries of the population aging process due to lifestyle changes, increasingly focusing on leisure compared to past generations [30].

### 2.3. Present-Day Development of Rehabilitation Engineering Tools to Satisfy Needs Related to Active Tourism

Design in rehabilitation engineering [29,34] is the intended planning of using assistive engineering devices (assistive technology, AT) in the targeted rehabilitation processes of people with disabilities. The development of assistive technology means and rehabilitation engineering involves numerous concepts and technical solutions facilitating participation in a variety of new leisure time activities for individuals with mobility issues, which use considerably exceeds their typical usage [35]. These may include, e.g.,

- Golf carts (for people with disabilities of lower extremities, making it possible to stand upright and preventing the risk of a fall);
- Wheelchairs adapted to travel on the beach and in the forest, equipped with electric engines and stabilized using electronic gyroscopes with multiaxial suspension, moving on cross-country tires or caterpillar tracks;
- Wheelchairs for mountain tourism, with an adequately reinforced frame and wheels, a low seat, with an electric or hybrid engine—a combination of the strength of human muscles and an electric drive—, with widely spaced wheels, frequently with additional handles facilitating pushing, pulling or carrying by assisting individuals). An example of such a vehicle is a special wheelchair designed for a mountain trekking trip to the Himalayas for “Michał Woroch—Himalayas Challenge_2021” [36];
- Equipment designed to facilitate sliding into the water when diving (e.g., with a walking mechanism rather than a wheel drive—according to patent [35]), special stationary equipment to transport individuals with motor disabilities from the shore or river bank to the water, equipment facilitating transfer from a wheelchair to a boat, kayak, and others;
- Alternative drive systems for manual wheelchairs enhancing their functionality (manual drives for hand cycling [37], the cam-thread drive as in patent application [38], electric or hybrid electric-manual assistive attachments to assist in covering longer distances and elevations [39]);
- Wheelchairs and electric or combustion engine vehicles to travel over boggy terrain (with multiple wheel or caterpillar track systems) [40];
- Adapted powered hang gliders—ultralight trikes and adaptations of planes with hand controls;
- Adapted sports cars for disabled drivers;
- Adaptations of cabin cruisers and sports yachts, e.g., specialist seats [41] and steering wheels for wheelchair users [42].

At present, thanks to the application of specialist technical solutions, many forms of tourism may be practiced by people with almost all types and degrees of disabilities. An example in this respect may be given by Hilary Lister, disabled due to muscular dystrophy, who in 2009 singlehandedly sailed around the British Isles. This was made possible thanks to applying a unique steering system using the sailor’s breath [43]. Another example is the 2018 trip through the North, Central, and South Americas (along a distance of over 65 thousand km) taken by Michał Woroch and Maciej Kamiński, who traveled in a sports utility vehicle with several adaptations for wheelchair users [44]. Still another example may be provided by the URI sailing yacht constructed in 1992, which may be used by unassisted wheelchair users [45]. For example, an innovative solution used there included specially designed access to the engine room of this yacht and the feasibility of inspections and minor repairs by wheelchair users.

Despite the dynamic development of assistive and rehabilitation technology solutions for various leisure time activities, it is still necessary to search for similar solutions assisting in tourism, including the use in the urbanized space [29]. These new solutions should include both individual technological solutions and infrastructure. They must provide new value or quality to achieve specific goals [46,47]. We may observe a high demand for new specialist assistive technology designs in currently relatively inaccessible areas of tourism, recreation, and sports, while at the same time, it is necessary to improve the quality of existing solutions. Such innovations are major objectives for the EU [48]. Eliminating barriers through legislation, adaptation, universal design, and other actions is the key to equal opportunities for people with disabilities. In Poland, the government program Dostępność Plus (Accessibility Plus) realized in 2018–2025 aims to support actions for people with disabilities. A significant area for action is connected with increasing transport accessibility for people with disabilities and senior citizens. The amount of PLN 23.2 billion (approx. EUR 5.4 billion) is allocated for support measures within the entire national program, of which PLN 20 billion (approx. EUR 4.6 billion) are intended for works related to the accessibility of transport, while PLN 900 million (approx. EUR 200 million) are allocated to services, including also barrier-free tourism and recreation. Within the support framework for services, it is planned to improve the accessibility of recreation areas, e.g., beaches and waterside areas, green areas such as parks and gardens, outdoor gyms and inclusive playgrounds, forest parking lots, and tourist attractions there or in their vicinity. Moreover, it is also planned to implement adaptive actions in tourist infrastructure (accommodation, trails, rental of specialist equipment for people with disabilities) and to improve the accessibility of tourist facilities and services (including, e.g., infrastructure of tourist hostels, resorts, and sanatoria, educational facilities of the State Forests or national parks, educational trails/nature trails, and tourist trails). The following results of work related to services are expected in Poland: providing a minimum of 100 km of tourist trails for people with disabilities, a minimum of 10 mountain shelters accessible for people with disabilities, 100 barrier-free cultural objects, as well as the creation of a system improving individual mobility for people with disabilities.

## 3. Case Studies of Innovative Devices

### 3.1. Selection of Cases for Analysis

The selection of cases of original practical and theoretical solutions is based on the decision to present innovative solutions, i.e., those that are far from obvious while being positive and novel, introducing new opportunities or expanding the functionality of previously known solutions. Such solutions include a self-propelled carriage platform with adjustable functionality, a drive mechanism for a wheelchair based on a completely novel principle of operation, and a yacht for disabled people and senior citizens and its selected equipment elements. They are examples of original concepts and equipment designs that meet the needs of mobility and tourism and rehabilitation of people with motor disabilities.

### 3.2. A Self-Propelled Carriage Platform for People with Disabilities

The necessity to support daily functions in various environments (the closed space of buildings and frequently non-adapted tourist spaces) requires the used means of rehabilitation engineering to exhibit an extensive and thus universal functionality (understood as a set of offered functions). To ensure functional mobility in all areas of life, a person with disabilities is forced to use a set of many specialized devices with limited and specialist uses (e.g., several wheelchairs adapted to specific applications, used by one person). The use of a specialist device outside its designed area of application may cause problems in the functioning, e.g., the drive of a wheelchair being of limited effectiveness outdoors, overloading of upper limbs and the shoulder girdle, the risk of injury, and problems with moving an all-terrain wheelchair in narrow and small spaces.

The need for excessive development of functionality in the case of a specific assistive technology device may lead to a deterioration of its function in many areas of its operation. An alternative may be provided by using an increasing number of various assistive means. However, their excessive number and the necessity to repeatedly select them to fit a specific situation is also far from advantageous.

**Figure 2:**
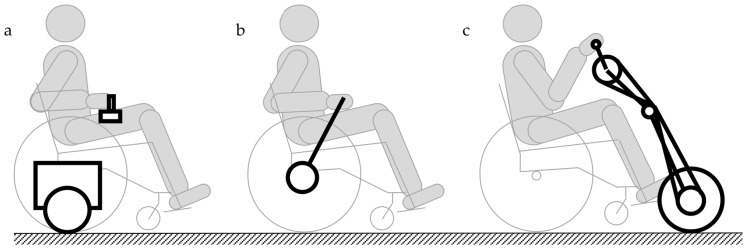
Changes to the drive mechanism of a manual wheelchair using: (**a**) an electric drive attachment, (**b**) a lever drive attachment, and (**c**) a handbike attachment.

It may also be assumed that a combination of various structural modules of currently produced devices, such as, e.g., a manual wheelchair and hand-bike attachments with electric drives (Figure 2), while disregarding potential adverse interactions, may lead to [49]:

- An uncontrolled overload of the wheelchair structure (attachments mounted at elements of the wheelchair structure at points that are not dedicated to such a solution);
- Damage to lacquer coating or at points of attachment mounting or damage to other elements;
- A lack of appropriate operation of combined elements or a lack of potential to combine them;
- Loss of performance properties of used products (e.g., it is impossible to safely sit down in a manual wheelchair with the mounted handbike attachment, altered seat geometry, deterioration of user comfort);
- Increased risk of injury.

Since expectations of potential wheelchair users are so diverse, it was assumed that designers need to consider developing designs for devices ensuring the simultaneous application of many functions. For this reason, a concept has been proposed for a family of transport or rehabilitation vehicles, which are structurally based on a common self-propelled carriage platform.

**Figure 3:**
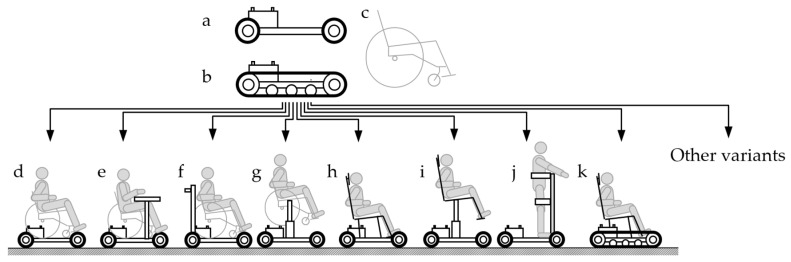
A modular, multi-variant system of means of transport for disabled persons: (**a**) a platform—a wheeled transport system, (**b**) a platform—an alternative caterpillar track transport system for the platform, (**c**) any manual wheelchair, (**d**) a transporter of a manual wheelchair, (**e**) a mobile work station, (**f**) a transporter adapted to being pushed by an assistant, (**g**) a transporter with a lifting system, (**h**) an electric wheelchair, (**i**) an electric wheelchair with a lifted seat, (**j**) a wheelchair with a standing device, and (**k**) an all-terrain caterpillar track transporter.

It was decided that the main structural element of the system will be the so-called self-propelled carriage platform used for independent transport of a sitting person indoors or outdoors without the need to use the muscle power of the human user. The platform has an alternative wheel or caterpillar track mechanism. It is adapted to the attachment of replaceable modules, e.g., aiding in standing upright, raising the seat, or providing specialist seats. The developed design [50] is a novel structure. It may be the basis for solutions of diverse forms and simultaneously serve a variety of functions (Figure 3). These are, for example:

- An electric wheeled transporter for a person with disabilities sitting in an active manual wheelchair (Figure 3d) for independent transport on roads, cycling paths, and in open spaces;
- An electric wheeled transporter with a study or work table for a disabled person sitting in a wheelchair (Figure 3e);
- An electric wheeled transporter adapted to transport a disabled person by an assistant (Figure 3f);
- An electric wheeled transporter with an additional lifting system for the manual wheelchair to increase the reach of the user’s arms in the workspace (Figure 3g);
- An electric wheeled transporter with a comfortable armchair securely supporting the body position, e.g., in the car, used for transport both outdoors and indoors (Figure 3h);
- An electric wheeled transporter with an electrically lifted comfortable armchair with an increased range of manipulation for shopping in supermarkets, residential and public buildings, and in urbanized areas (Figure 3i);
- An electric wheeled transporter, with a standing device to verticalization body position, which is especially needed by people sitting in wheelchairs (Figure 3j);
- An electric caterpillar track transporter with a comfortable seat providing secure support for the body using seatbelts for driving in non-urbanized areas for tourism and recreation (Figure 3k).

As a result of Figure 3 and previous descriptions, the self-propelled carriage platform is the basis for the modular system composed of several functional modules. This platform is used to develop families of new transport and rehabilitation means, which may be used outdoors. The platform of this system is a specialist wheelchair with an electric drive, which can be used without assistance (or with partial assistance). Periodical use of modules (e.g., a recreational drive, a cross-country trip, or in the education institution context—for classes) results from the simultaneous use of a manual wheelchair over short distances. The described platform needs to fit inside the trunk of a passenger car. The manual wheelchair user can transfer to and from the platform without assistance.

**Figure 4:**
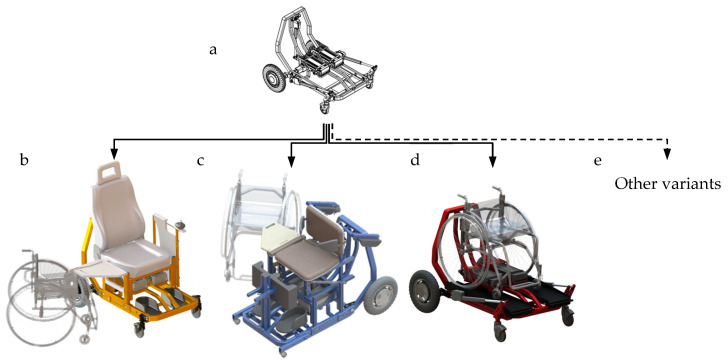
A platform and selected representative types of the designed family of devices (based on [46]): (**a**) the platform, (**b**) an electric wheelchair, (**c**) the wheelchair platform, (**d**) an electric verticalization system, and (**e**) other solutions designed using the transport platform.

The structural design of the platform and three selected designed representative types of the designed family are shown in Figure 4.

The presented solution is based on a typical modular approach used in technology (e.g., one car floor panel used in the construction of many car types). Designing modular structures requires high structural and economic inputs. However, the rationalization effect results from a combination of pre-manufactured modules (parts or assemblies) providing the desired variant of a technical function. The modular segment structure is a system of assemblies and parts, which are modular segments that perform various general functions of the system [51]. In this case, the platform yields more functional options. Having several attachments and their simple assembly on the self-propelled carriage platform ensures their alterations depending on the operational needs of a person with disabilities.

Such a solution is advantageous compared to a set of many single-function AT devices while simultaneously answering the demand for products characterized by atypical functionality. The basic structure of the transport platform facilitates the manufacture of vehicles of different functions at reduced production costs. The simple structure is provided, among other things, by applying the produced drive and steering systems. The solution has universal applications, both outdoors and indoors. It is operated with no assistance needed.

This solution makes it possible to participate in various forms of activity in urban and adapted non-urban areas. At the application of the caterpillar tack transport system, it may be used in a non-adapted terrain and allow the user to involve in tourism. The application of the structure may promote increased physical activity, thus promoting rehabilitation. Despite the use of an electric drive (which may be associated with a limitation to physical activity), it expands the range of travel in the therapeutic sense. At the same time, within the scope of rehabilitation, it results in mobilization to rest in non-urbanized surroundings and the transfer, balance, and stabilization of the body while scaling terrain obstacles such as elevations and slopes, traversing obstacles, or promoting more active interactions with the surroundings as well as easier transport and performance of daily life functions.

### 3.3. The Cam-Thread Drive for a Wheelchair

**Figure 5:**
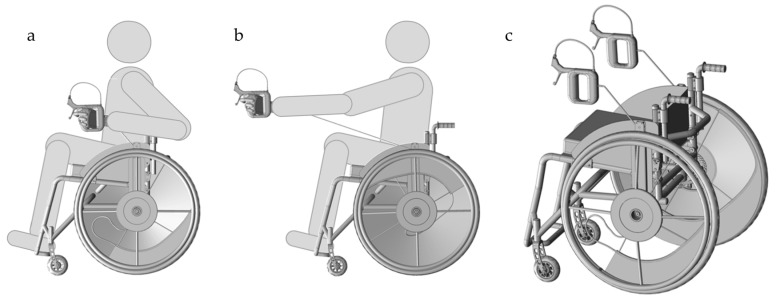
A wheelchair with the cam-thread drive: (**a**) the initial sequence of the drive, (**b**) the final sequence of the drive, and (**c**) axonometric view.

A typical drive for wheelchairs using drive rings is very simple and intuitive although burdened by several drawbacks. One of them is permanent, determined by the wheelchair structure, a trajectory of the drive motion of upper limbs [52,53]. The cam-thread drive of a wheelchair, shown in Figure 5, is an addition to a typical wheelchair with a manual drive, which facilitates a change in the drive method [38]. The described innovative drive is composed of two mechanisms, with one for each wheel of the wheelchair. The thread (string), to which the drive handle is attached, is flexible, inextensible, and facilitates the drive motion in any direction, e.g., abduction, extension sideways, and extension downwards. The rectilinear drive motion of the string is transferred into the rotational motion of the wheel using the cam disk.

**Figure 6:**
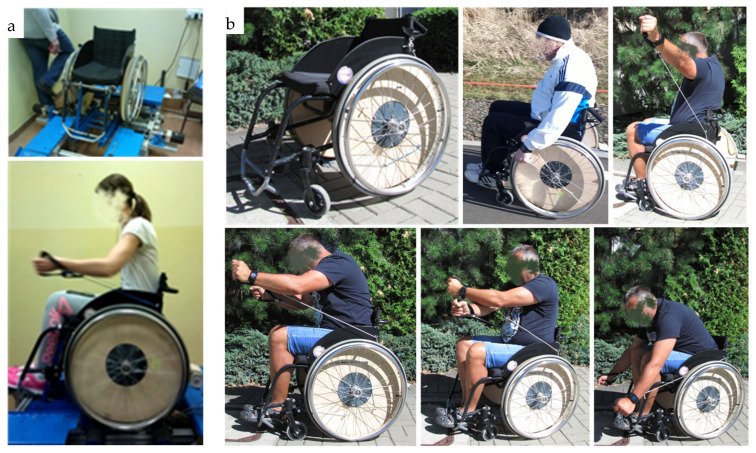
A prototype solution for a cam-thread drive wheelchair: (**a**) tests at the test bench and (**b**) field test.

Wheels with the additional cam-thread drive may be mounted using a unified quick coupler to any manual wheelchair to replace typical drive wheels with drive rings. The application of cams with “progressive” characteristics (i.e., those with a decreasing radius) makes it possible to obtain a changing gear ratio for the wheelchair drive. When the wheelchair starts moving, a large motion of the string causes a small rotation of the wheel with large torque; next, the rotational speed of the wheel increases, facilitating faster travel. Driving the wheelchair only using short motions performed with high frequency slows the speed of travel and makes it possible to traverse a slope; in contrast, driving the wheelchair applying long motions of lesser frequency leads to a faster speed of travel. This type of drive works well over longer distances covered outdoors. Indoor mobility is slightly more difficult [54]; however, leaving the typical drive rings on wheels ensures the conventional drive method for the wheelchair when required. The wheel with the cam-thread drive may be an attachment, which provides a drive for the wheelchair while simultaneously being a device for strength training or rehabilitation. The application of maneuvering elements in the form of an elastomer spring for tubing exercise enhances the rehabilitation value of this drive. The opportunity to perform movements with upper limbs in various directions promotes rehabilitation and activation of different groups of muscles in the upper limbs and the shoulder girdle. The tests (Figure 6) indicate the considerable potential of this drive.

### 3.4. A Marine Yacht for People with Disabilities

Various attempts have been made to adapt vessels for the needs of people with disabilities and senior citizens. Despite the superficially similar structure resembling conventional yachts, such vessels are considerably more varied in terms of their design and structure. The first problems with the adaptation of the hull of a traditional motorsailer for the needs of people moving on wheelchairs are experienced already at the designing stage. The deck needs to be widened, thus changing the geometric proportions of the yacht to facilitate the movement of disabled people in wheelchairs. A separate, huge design task when producing a yacht for disabled sailors is connected with considering different types of disabilities. Basic assumptions for the vessel design include the following:

- The size of the yacht ensures simultaneous long-distance travel for 10–16 people, including a maximum of 4 people moving on wheelchairs;
- The potential for unassisted sailing by senior citizens and people with disabilities independent of non-disabled people;
- Sailing by people with diverse disabilities (optimally for people with different motor, sensory, and mental disabilities as well as those with aging-related dysfunctions traveling together during the same cruise);
- Minimization of yacht size/length (e.g., to reduce fees paid in marinas);
- People with disabilities may perform sailing operations (sail handling, mooring, cooking meals);
- Large, safe, easily accessible open surfaces, e.g., the upper deck superstructure;
- The yacht is equipped with means supporting accessibility for people with disabilities first of all when walking, standing, sitting down, grasping, holding, orientation and identification of space, and receiving information.

Developing a set of requirements for a yacht intended for people with disabilities is difficult because of the design and structure complexity. For example, the large surface area of the yacht promotes the comfort of operations and staying and moving inside the yacht. However, it is not advisable to considerably enlarge the yacht. Minimizing the size of the yacht results from the need to reduce the maintenance and servicing costs of the vessel. Frequently, an excessively large surface, e.g., of the Kingston valve, may lead to a risk of instability and bumping into the device or a fall in the case of disabled sailors. In turn, a too-small yacht may prevent the application of all required accessibility and safety measures. Negotiating the requirements and optimizing the multiple criteria determining the yacht size is necessary. A yacht for people with disabilities needs to include several different technical solutions compared to conventional vessels. Adopted technical solutions should ensure the potential for unassisted sailing independent of non-disabled people. An important aspect when assuring safety on the yacht is to apply various safety systems and to develop procedures for the performance of operations under different weather conditions and in various situations. The yacht should be equipped with special devices such as yacht wheelchairs for individuals whose mobility limitations (not necessarily paraparesis) may increase the risk of accidents under changing sailing conditions. Designing a yacht wheelchair is a new task. A yacht wheelchair may be used as an alternative to personal wheelchairs by people with disabilities. It will provide enhanced functionality compared to personal wheelchairs.

It is assumed that yacht proportions are approx. 3:1, and they determine the *L*_c_: *B* ratio (total length to width) of the yacht hull amounting to approx. 18:6 m, which are atypical proportions compared to conventional designs of marine sailing boats. These proportions, primarily the considerable width, ensure accessibility of decks for people with disabilities moving on wheelchairs and using other rehabilitation devices. The increased yacht width facilitates free movement and provides an increased area of facilities (e.g., the toilet, the forecastle, space for wheelchairs next to berths) and passageways/traffic routes as well as a functional superstructure of min. 2 m usable height. The provided yacht dimensions will be the requirements defined as obligatory when presenting further considerations for yacht accessibility.

As mentioned above, despite the high potential health risk when practicing such a form of physical activity, yachting brings several benefits to disabled people, as mentioned above. It positively impacts the process of social rehabilitation, including elements of rehabilitation enhancing physical fitness. Adaptation of yacht elements to the needs of people with motor disabilities requires several alterations in terms of the procedures and technical means assisting in mooring, handling the sails, the steering wheel and the anchor, moving around the yacht, physiological needs, as well as main material aspects of living conditions (e.g., available berths).

**Figure 7:**
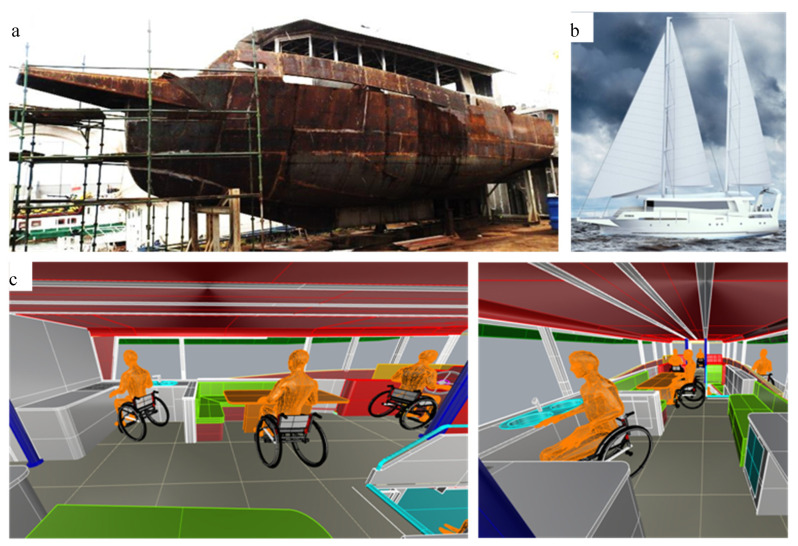
A yacht for disabled people: (**a**) the yacht under construction, (**b**) yacht visualization [45], and (**c**) design tests (analysis of yacht superstructure ergonomics: the galley and the messroom).

Currently, the authors are designing the described A-class (ocean-going) yacht (under the working name: Empatia 60 FD—For Disabled) adapted to the needs of people with all types of disabilities. Figure 7 shows the hull of the constructed yacht and its computer visualization.

Because of the increased interest in various leisure activities and active participation in sports by senior citizens and people with disabilities, an important problem has been observed in yachting with the adaptation of the pilothouse or the steering wheel station to the needs of people sitting in wheelchairs or other seats (e.g., due to their age older people are unable to stand upright over extended periods). In the case of a conventional steering wheel, it is a problem to reach the wheel when sitting down comfortably. The knees limit the access to the steering wheel, making the operation uncomfortable and causing strain in the long run (excess physical effort, an uncomfortable body position). As a rule, a classical large-sized steering wheel has a handle in the form of a large ring. The large diameter of this ring results from the need to provide an adequate gear ratio in traditional solutions with a rudder gear. The large dimensions of the steering wheel are also justified by the historical reference of the steering wheel design to the sailing traditions, which even today has an impact on the use of such a structural element. However, these are solutions dedicated to able-bodied operators in standing positions.

People sitting in a wheelchair can use a typical large steering wheel when positioned at a certain distance from the steering wheel; however, it is not an entirely satisfactory or ergonomic solution. Literature on the subject presents only scarce examples of solutions to improving the quality of use of a steering wheel by people with disabilities and senior citizens.

Other solutions are available for small steering wheels for individuals when sitting down. The small-sized steering wheel (resembling the car steering wheel) is mounted similarly to that in cars (e.g., yachts: Uri [55], Impossible Dream [56], yacht as presented in the patent application CN 206,068,111 U [57] or the invention of the steering wheel as presented in the patent application CN 203,902,833 U [58]); in this case, it is positioned in the comfort zone for the arms of the person steering. These are solutions replacing a typical large steering wheel. An adequate gear ratio and the power-assisted steering between the steering wheel and the helm ensure the comfortable steering of yachts. Such solutions have been implemented on yachts, on which people with disabilities may serve as crewmen.

**Figure 8:**
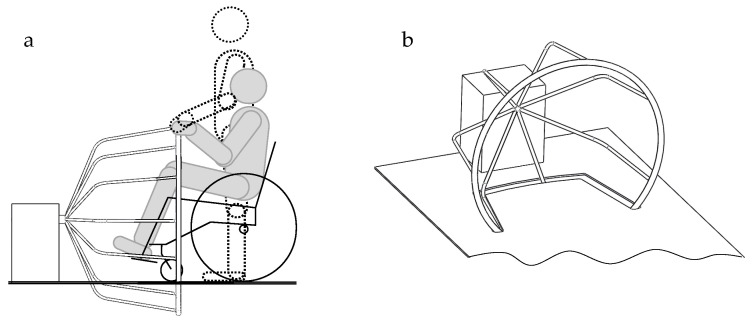
A diagram of the steering wheel solution, following the principles of universal and transgenerational design: (**a**) a steering wheel with silhouettes of a person standing and a person sitting in a wheelchair and (**b**) the axonometric view of the solution.

Applying a large steering wheel for people with disabilities requires redesigning this solution. This is done, e.g., using wheel arms of molded shape, providing space for the front part of the wheelchair or any other seat and the lower limbs of the sitting person [42] (Figure 8). It is essential to design a large (full-sized) steering wheel, which promotes the comfort of use due to a large section of the exposed steering wheel diameter (in contact with the hand of the disabled person) when the wheel is being turned. The steering wheel dimensions ensure comfortable steering operations, as it is possible to reduce the distance between the body axis and the steering wheel (steering does not have to be made using extended arms) and to take the central position at the wheel. The arms of the steering wheel on the opposite side are attached to the steering wheel axis, which joins the steering wheel column on the deck, and the deck has a bushing groove for some steering wheel parts and arms.

A yacht, as a craft dedicated to people with disabilities, may promote activization in life and cooperation in yacht handling operations on the deck while the crew is staying together during extended cruises may initiate contacts and human interactions. The opportunity to perform handling operations on an adapted yacht provides a sense of being needed while enhancing psycho-physical fitness. The yacht’s swaying and tilting are challenging; they enforce the need to maintain body balance, improve the sense of balance, and promote increased attention. A marine yacht is first of all a means for the comfortable practice of tourism. It is an element fostering interest in travel and increasing life activity in senior citizens and people with disabilities.

## 4. A Proposal for an Algorithm for the Design of Innovative AT Devices

**Figure 9:**
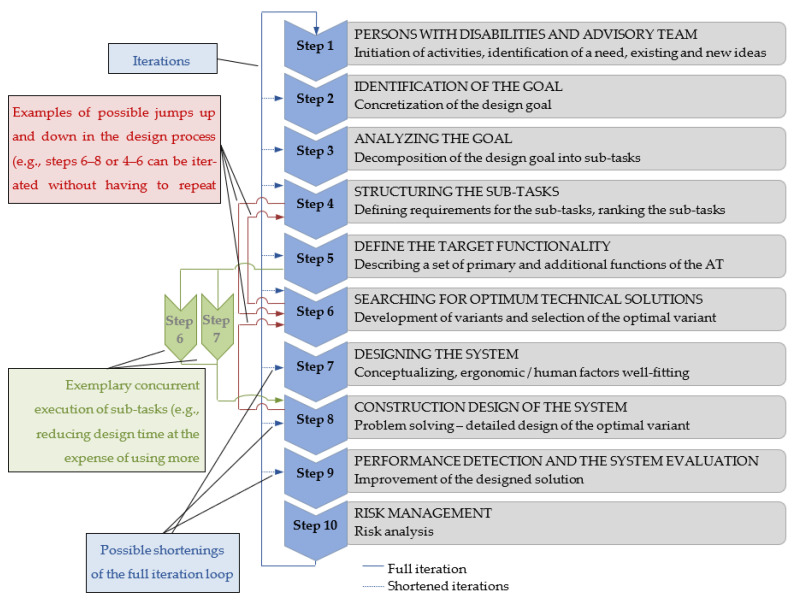
A model of the design process for assistive technology (AT) for people with disabilities (based on [61]).

Assistive devices’ design combines technical and social knowledge (the knowledge of therapeutic needs and occupational performance) to gather a user-centered approach [59]. Development and adaptation of assistive technologies to the needs of a specific disabled person may be presented in the form of an algorithm. Algorithms are available for the adaptation of assistive technology devices dedicated to meeting the needs of a specific user. For example, Federici et al. proposed four steps of measurement and assessment of the algorithm: the “user data collection” stage (step 1), reading and interpreting the data by a multidisciplinary team (step 2), the “matching process” (step 3), and the “follow-up” stages (step 4) [60]. This algorithm facilitates an optimal adaptation of a set of assistive technology devices to the needs of any disabled person based on the person’s description using the International Classification of Functioning. However, it is challenging to design novel assistive technology devices that expand the typical areas of functioning for a person with mobility limitations [61]. Based on our experience in this respect, the authors would like to propose a 10-step algorithm for the process, as shown in Figure 9.

The proposed algorithm starts with the identification of needs (step 1). Adequate identification of needs for the object of design is the basis for initiating actions in the AT design process. It facilitates an appropriate identification of the design goal (step 2). Designing to meet the needs of disabled people or senior citizens needs to be a human-centered design of an optimum system consisting of “a person with disabilities—assistive technology—environment of its operation”. Human-centered design (HCD), also known as user-centered design (UCD), is a methodology for designing systems well-fitted to their users. According to ISO 9241-210:2019, human-centered design is a design philosophy and process in which the needs and limitations of users influence the different stages of the design process [62]. At HCD, the emphasis is on the ability to predict how users can and will use the product and what testing and behavior prediction tools are most beneficial to users in the real world. For this reason, HCD is an excellent method to break down the design goal into sub-tasks (step 3).

The next step is to parametrize individual tasks (step 4), i.e., to determine their boundary and expected parameters expressed in physical units, e.g., admissible dimensions of the designed device, its weight, velocity, range, etc. At this stage, technical discrepancies in the designed device are identified. This requires the selection of primary and auxiliary functions of the device (step 5). The set of these functions defines the overall functionality of the device. Not all device functions are equally important, as some functions may adversely affect others. For example, excessive parameters of the function of the user’s body stabilization in the wheelchair result in a deterioration of the wheelchair’s transport function. For this reason, it is necessary to order the importance of functions of the designed system of “a person with disabilities—assistive technology (AT)—environment of its operation” and to improve the quality of the AT primary functions even at the expense of elimination or deterioration of certain auxiliary functions. Simultaneously with the selection of primary and auxiliary functions for the designed AT device, the process of searching for optimum technical solutions takes place (step 6). Selection of the structural design variant makes it possible to design a system ergonomically well-fitting the designed system of devices to a human user (Step 7). Ergonomic design (or “human factors” design, as it is referred to in the USA) is a framework for designing or arranging products and systems (workplaces) so that they are comfortable for the people who use them [63,64]. It is essential when designing AT since people with disabilities are usually more sensitive to non-ergonomic solutions than non-disabled individuals [65,66,67,68]. This imposes the need for a multifaceted analysis of the object’s limitations due to disability and frequently also when considering the adaptation of the product to the ability to perform the physical tasks of daily life (physiological functional capacity, PFC).

Detailed design solutions (step 8) comprise a thorough selection of specific structural design solutions. Limitations in this selection are connected with the production capacity, availability of standardized parts, and structural properties of used materials. Other types of specific requirements are also encountered. For example, when designing assistive technology solutions for tourism of people with disabilities, it is necessary to predict the impact on these products exerted by various atypical environmental conditions, such as seawater, considerable fluctuations in temperature, strong wind, swaying of the yacht deck, considerable vertical accelerations of the yacht deck at wave motion, or the impact of rain. Sea water causes significant problems in using durable structural materials; it may also lead to injury (in the case of people moving in wheelchairs, the water drying on the wheelchair seat and the formed salt residue may cause skin laceration). In turn, wind and swaying may result in less stability for people moving around the yacht; thus, it is vital to eliminate the risk of being hit by designing, e.g., foot shields for people moving on wheelchairs (due to spinal cord damage, foot numbness, and no sensation of pain, accidental bumps may be very dangerous for users [69]), and the impact of air temperature at high humidity may lead to hypothermia, etc. Considering the environment’s specific character is essential in product design [70].

The designed system requires evaluation (Step 9). Evaluation consists of the verification of the actual functionality of the designed system compared to that planned in the design. An obligatory element is to conduct verification of product consistency with legal regulations. These requirements refer to the set of specifications and technical standards applicable to a specific type of device. A frequent problem in the design of innovative devices is related to the lack of respective standards and regulations. It is necessary to assimilate and apply the requirements imposed on products with comparable functions or to develop their standards in such a situation. Before implementing the designed AT device or system, the final step consists of the risk analysis for their use. Risk analysis methods are well-standardized and described in literature on the subject [71].

When reviewing known models of the engineering design process (see Rohatyński [72] and later models, e.g., the evolutionary model [73], the Munich procedural model [74], or the development of mechatronic systems according to VDI 2206) it may be stated that they may not be easily transformed to designing products for disabled people. The development of assistive technology and rehabilitation engineering devices requires the participation of people with disabilities in the design process (e.g., [75,76]). Defining the utility and performance requirements may be done in many different ways, e.g., by analyzing users’ needs and assessing the requirements imposed on designed structures by the designer; this form of assisting design activity seems indispensable to ensure the development of well-adapted AT products [77].

The presented original design process model (Figure 9) is based on known design procedures and focuses on the user and the systemic approach. It is assumed that the co-participation of users in the product design takes place at all steps. Information is obtained from users using many methods. The type and number of applied methods to investigate users’ requirements may change depending on the needs of the presented design task and its solution. In addition to AT users, the interdisciplinary design team should also include assistants of disabled people and other decisionmakers.

The process presented in Figure 9 is sequential. It allows multiple repetitions of each step in successive iterations leading to a specific solution. The number of iterations and the scope of design actions at each step depend on the nature of the AT type being designed. At each step, known systemic methodologies supporting the design process may have to be applied. In each of the ten steps of the design process, a characteristic cycle of procedures may be identified: (1) definition of the task, (2) formulation of the assumption—hypothesis, (3) creation of the solution variant, (4) verification of the hypothesis, and (5) results of the solution. Actions within the process may also be unconditional (any reasons for selection do not limit them) and conditional (executed considering conditions resulting from other limitations of the process).

Application of the proposed design process makes it possible to solve diverse design problems. Starting from the formulation of the task through the presentation of a hypothesis, the successive variants of solutions may be transformed and construed, while the hypotheses may be verified, and the results may be obtained.

## 5. Conclusions

Developing designs for assistive mobility technologies should lead to an extension in the forms of participation in various activities among senior citizens and people with disabilities, particularly in tourism and sports, which until recently were considered less in comparison to such needs as, e.g., all potential activities in the urbanized space. Presently, the development of assistive technology (AT) measures comprises specialist equipment, facilitating participation in various leisure activities including active tourism. Innovative AT devices are frequently designed and manufactured in single units and commissioned to be custom-made to meet the needs of a small number of people. On the one hand, the development of rehabilitation engineering devices aims at improving the functionality of available equipment, which has been redesigned many times before (e.g., manual wheelchairs) to improve the quality of their use in spaces adapted to their usage (indoor spaces, urban spaces). However, the aim is to find solutions for the designed devices to expand the area of their applications (various forms of active tourism and sports). In the case of the first of the mentioned groups, the structural modifications are small changes introduced in the multi-stage design iterations, and as a result, their performance parameters are slightly upgraded; nevertheless, they are still very important. In the second group, we may expect innovative AT solutions with novel, frequently only partly verified applications. For example, in sports disciplines previously unavailable for people with disabilities, the newly developed technical means make it possible for such people to practice these sports.

Nevertheless, they are typically based structurally on models of known solutions, e.g., the design of frames for typical wheelchairs used in adapted sports, fitness, or leisure activities or yachts for disabled people. The technical solutions presented in this paper are only selected examples of such designs. This field is and will continue to be the subject of further design works on innovations aiming at improving life in the integrated society.

The presentation and analysis of original, innovative concepts for technical solutions dedicated to people with motor disabilities as well as the subjective evaluation of progress in the development of technologies for people with motor disabilities constitute the basis for the proposed design process algorithm focused on the needs of people with disabilities. The proposed algorithm makes it possible to create new classes of assistive technology devices and opens new areas of activity for people with significant disabilities, which supports the maintenance of mental and physical well-being in different groups of disabled people in the long-term perspective.

## Data Availability

All data generated or analyzed during this study are included in this published article.
